# Altered PI3K/mTOR Signaling within the Forebrain Leads to Respiratory Deficits in a Mouse Model of Epilepsy

**DOI:** 10.1523/ENEURO.0292-25.2025

**Published:** 2025-12-16

**Authors:** Patrick Woller, Vamshidhar Singidi, McKenzie Rice, Durgesh Tiwari, Christina Gross, Steven A. Crone

**Affiliations:** ^1^Neuroscience Graduate Program, University of Cincinnati College of Medicine, Cincinnati, Ohio 45267; ^2^Division of Neurosurgery, Cincinnati Children’s Hospital Medical Center, Cincinnati, Ohio 45229; ^3^Department of Medical Sciences, University of Cincinnati College of Medicine, Cincinnati, Ohio 45267; ^4^Division of Neurology, Cincinnati Children’s Hospital Medical Center, Cincinnati, Ohio 45229; ^5^Departments of Pediatrics, University of Cincinnati College of Medicine, Cincinnati, Ohio 45267; ^6^Neurosurgery, University of Cincinnati College of Medicine, Cincinnati, Ohio 45267; ^7^Division of Developmental Biology, Cincinnati Children’s Hospital Medical Center, Cincinnati, Ohio 45229

**Keywords:** breathing, diaphragm electromyography, epilepsy, forebrain control of breathing, mTOR pathway, sudden unexpected death in epilepsy

## Abstract

People with epilepsy may experience sudden death due to respiratory failure through mechanisms that are currently not well understood. Epilepsy causing mutations are thought to elicit seizures due to altered function of forebrain circuits, yet breathing is controlled largely by the brainstem. To investigate how altered forebrain activity could impact breathing, we examined respiratory and seizure phenotypes in a mouse epilepsy model with a forebrain-specific deletion of the phosphatase and tensin homolog (*Pten*) gene. Using chronic diaphragm electromyography and cortical electroencephalography, we monitored *Pten* conditional knock-out (PTEN–cKO) mice (six males and four females) and control littermates (six males and three females) continuously from preseizure onset through end-stage disease. PTEN–cKO mice develop spontaneous seizures that progress in frequency with age, accompanied by gradual changes in respiratory function, even during interictal periods. As seizure burden increases, PTEN–cKO mice experience an increased frequency of interictal apneas, slowing of respiratory rhythm, prolongation of inspiratory bursts, and elevation of inspiratory effort. All animals experienced a terminal apnea prior to cardiac arrest. These findings demonstrate that *Pten* deletion in the forebrain disrupts the control of breathing and leads to terminal respiratory failure.

## Significance Statement

Epilepsy typically alters forebrain function, but whether this alone is sufficient to alter the control of breathing, particularly in the absence of seizures, remains unclear. Here, we show in a spontaneous seizure model that forebrain-specific mutation of phosphatase and tensin homolog leads to progressive respiratory abnormalities initially manifesting as increased interictal apneas, then progressing to slower, deeper breathing and ultimately terminating in respiratory failure. These findings suggest that forebrain circuit dysfunction in epilepsy can disrupt respiratory control, potentially increasing the risk of sudden unexpected death in epilepsy.

## Introduction

Phosphatase and tensin homolog (PTEN) is a lipid phosphatase and well-established tumor suppressor that plays essential roles in cellular homeostasis (Verrastro et al., 2016; Chen et al., 2018; Shan et al., 2019; Skelton et al., 2020). In the central nervous system, PTEN modulates the neuronal size, synaptic plasticity, axonal arborization, and network excitability (Fraser et al., 2008; Sperow et al., 2012; Haws et al., 2014; Dusing et al., 2023). Conditional knock-out (cKO) mouse models with region- or cell type-specific deletions of the *Pten* gene have revealed its necessity for maintaining proper cortical architecture and circuit dynamics (Backman et al., 2001; Sperow et al., 2012; LaSarge et al., 2021). Disruption of PTEN signaling in the brain has been linked to multiple neurodevelopmental and neuropsychiatric disorders, including macrocephaly, autism spectrum disorder, and epilepsy (Zhou and Parada, 2012; Lim and Crino, 2013; Rademacher and Eickholt, 2019; White et al., 2020). In particular, mutations in PTEN and other mammalian target of rapamycin pathway regulators are frequently observed in drug-resistant epilepsies and cortical dysplasias, in both syndromic and nonsyndromic forms (Galanopoulou et al., 2012; Nguyen et al., 2015; Matsushita et al., 2016). In both human patients and mouse models, loss of PTEN in forebrain neurons leads to spontaneous seizures, behavioral abnormalities, and premature lethality (Pun et al., 2012; White et al., 2020; Koboldt et al., 2021). The mechanisms underlying PTEN-related epileptogenesis include an imbalance of excitation and inhibition, enhanced synaptic protein synthesis, and aberrant neuronal connectivity (Gross and Bassell, 2014; Santos et al., 2017; White et al., 2020). However, while the impact of PTEN deletion on cortical excitability is well documented, its influence on brainstem-regulated physiological processes, such as breathing, remains poorly understood (Zhan et al., 2016).

In healthy animals, cortical and subcortical circuits interact with brainstem respiratory networks to adapt breathing patterns to behavioral and environmental demands (Pattinson et al., 2009; Zoccal et al., 2014; Sheikhbahaei et al., 2020; Schottelkotte and Crone, 2022). This can occur through direct or indirect connections from the forebrain to respiratory rhythm generating nuclei in the medulla, including the pre-Bötzinger complex, parafacial respiratory group, and the postinspiratory complex (Schottelkotte and Crone, 2022). In epileptic brains, however, this modulatory control may become maladaptive. Seizures can disrupt respiratory rhythm generation, induce postictal apnea, and interfere with arousal mechanisms (Buchanan and Richerson, 2010; Dlouhy et al., 2015; Zhan et al., 2016; Nobis et al., 2019). Recent studies have shown that mutations confined to cortical neurons, such as those in DEPDC5, can nonetheless produce fatal breathing abnormalities in mice, including ictal or postictal apneas and respiratory failure (Kao et al., 2023). These findings demonstrate that forebrain dysfunction can lead to impaired breathing, consistent with our results using PTEN–cKO mice.

Respiratory monitoring is rarely performed in epilepsy patients outside of the hospital. It remains uncertain whether respiratory abnormalities are confined to seizure episodes or also manifest during interictal periods—which could potentially serve as a biomarker for sudden unexpected death in epilepsy (SUDEP) risk. This study seeks to address this gap by employing chronic electroencephalography (EEG) and diaphragm electromyography (EMG) recordings in PTEN–cKO mice to evaluate the impact of forebrain circuit dysfunction on respiration both during and outside of seizure events.

In this study, we demonstrate that selective deletion of PTEN in forebrain neurons leads to progressive and ultimately fatal respiratory dysfunction. Using continuous EEG and diaphragm EMG recordings, we found that PTEN–cKO mice exhibit increased interictal apnea frequency, slowed breathing, and increased inspiratory effort, particularly during NREM sleep and late-stage disease. These respiratory disturbances emerge as seizure frequency escalates and become more severe after the onset of persistent epileptiform activity (PEA), culminating in terminal respiratory arrest that occurs before cardiac failure. Our findings show that cortical circuit dysfunction can perturb breathing, even during interictal periods, and ultimately lead to respiratory failure.

## Materials and Methods

### Animal models

*Pten^fl/fl^* (“floxed”) and *Tg^CamKIIα-Cre/+^* mice were obtained from Jackson Laboratory [B6;129S4-PTENtm1Hwu/J, JAX #006440; and B6.Cg-Tg(CamKIIa-cre)T29-1Stl/J, JAX #005359]. All mouse lines were maintained on a C57BL/6J background, consistent with the background of the original JAX strains. Experimental cohorts were composed of littermates to minimize background-related variability, as in our previous study using this line (White et al., 2020). PTEN–cKO mice (*Pten^fl/fl^*; *Tg^CamKIIα-Cre/+^*) and their littermate controls were generated by crossing male *Pten^fl/+^*; *Tg^CamKIIα-Cre/+^* with either female *Pten^fl/+^* or *Pten^fl/fl^* mice. Littermate controls in this study were either *Pten^fl/fl^*; *Tg^+/+^* (*N* = 5) or *Pten^fl/+^*; *Tg^+/+^* (*N* = 4; Tsien et al., 1996). No significant differences in apnea frequency^a^ or respiratory rate^b^ was detected between these subgroups; therefore, they were pooled for the main analyses presented here. We did not include* Pten^fl/+^*;* Tg^CamKIIα-Cre/+^* or *Pten^+/+^*;* Tg^CamKIIα-Cre/+^* control cohorts, which also do not exhibit seizures (McMahon et al., 2012; White et al., 2020). Experimental *Tg^CamKIIα-Cre/+^*;* ROSA^PnP-tdTomato/+^* and littermate controls were generated by crossing male *Tg^CamKIIα-Cre/+^* with *ROSA^PnP-tdTomato/+^* [Jackson Laboratory, Ai14 line, B6;129S6-Gt(ROSA)26Sortm14(CAG-tdTomato)Hze/J, JAX #007908] females. Mice were genotyped via polymerase chain reaction.

Mice were housed in standard cages (up to four per cage) on a 14:10 light/dark cycle with food and water provided *ad libitum*. Mice implanted with transmitters were singly housed. All procedures were approved by the Institutional Animal Care and Use Committee and complied with the Guide for the Care and Use of Laboratory Animals.

### Telemetry device implantation surgery

Telemetry devices (HD-X02; Data Sciences International) were implanted in PTEN–cKO and control mice as early as 6 weeks of age to enable chronic recording of diaphragm EMG and cortical EEG. Surgeries were performed on mice between Postnatal Day (P)42 and P51 and at a minimum body weight of 20 g. In 8/10 PTEN–cKO and 7/9 control littermate animals, the transmitter was implanted in the intraperitoneal cavity as follows. Under isoflurane anesthesia, mice were placed supine, and an approximate 3 cm lateral incision was made through the abdominal wall just below the rib cage. The xiphoid process was clamped with hemostats to expose the diaphragm. The transmitter was positioned in the left abdominal cavity adjacent to the intestines. One set of leads was reserved for cortical placement, while the other was positioned near the diaphragm. Forceps were used to gently lift the right costal diaphragm, and the exposed wire of the positive lead was inserted through the first muscle layer without puncturing the second. Extreme care was taken to ensure that the diaphragm was not punctured. The exposed wire was secured in place against the diaphragm muscle with a small drop of surgical glue (GLUture, Zoetis). The negative lead was inserted in the same manner, ∼1 mm dorsal to the position of the positive lead. Excess lead length was coiled and placed just below the abdominal muscle and above the implanted transmitter. The abdominal muscle was closed over the transmitter and secured with sutures, with care taken to externalize the second pair of biopotential leads for cortex insertion. The skin incision was closed using inside-out sutures, leaving ∼1 cm open closest to the mouse's head where the second set of leads remained exposed. A second incision was made between the mouse's shoulder and ear to expose the trapezius muscle. A trocar was used to tunnel the second set of exposed leads subcutaneously from the intraperitoneally implanted transmitter to the open incision near the mouse's shoulder. In a subset of animals [two control and two PTEN–cKO mice (Animals B and C)], the transmitter was instead implanted subcutaneously on the back rather than in the abdomen. In these cases, a smaller abdominal incision was made to access the diaphragm. Using a trocar, the diaphragm leads were tunneled subcutaneously from the back-mounted transmitter to the open abdominal cavity, where they were affixed to the diaphragm as described above. Excess lead length was coiled and positioned within the abdomen prior to closure. The mice were then placed on the stereotaxic frame and received maintenance anesthesia at 1–2% isoflurane. Two burr holes were drilled (AP, −2.5 mm; ML, ±2 mm from the bregma) without penetrating the dura. The positive and negative transmitter leads were inserted into the burr holes to contact the dura and fixed into place with Ortho Jet Liquid Powder Kit (Patterson Dental). The incision was closed with suture (Covidien), surgical glue, and treated with antibiotic ointment (RARO). Mice were placed in a 30°C incubator to recover overnight. Mice were allowed to recover for at least 3 d before any data acquisition.

### Acquisition of diaphragm EMG, cortical EEG, and video

Mice were individually housed in a clear home cage placed on top of a receiver that continuously and wirelessly recorded diaphragm EMG and cortical EEG. Animals had *ad libitum* access to food, water, and ambient air throughout the recording period. Video was recorded simultaneously using cameras mounted in front of each cage to enable behavioral monitoring and synchronization with electrophysiological data. Long-term recordings were conducted 24/7 until PTEN–cKO mice died ∼9–11 weeks of age. Littermate controls were killed when their littermate PTEN–cKO mice had died.

### Analysis of EEG to detect seizures

Seizure activity was assessed using the Spike2 software (Cambridge Electronic Design; RRID:SCR_000903; Spike2 script SleepSpindle/Seizure Detection). Seizures were defined as abrupt increases in frequency and amplitude (>2 times the baseline) lasting longer than 10 s (Fisher et al., 2005; Johnson, 2019). Because EEG was recorded only from the cortex, our analysis is limited to seizures that propagated to or originated in cortical regions. Identified seizures were verified with video recordings and Racine scale analysis (Racine, 1972). We used seizure frequency to define stages of disease progression (0, 1, 2–3, 4–5, 6–10, and 11–20 seizures per day) to compare breathing between mice at similar stages of disease progression.

The duration of PGES was examined with the Spike 2 (Cambridge Electronic Design) software. A moving window (5 s) determined the duration of PGES events. The end of each PGES event was determined by taking the mean EEG power of preictal baseline (100 s), subtracted from the mean of the moving window until postictal EEG power exceeded three standard deviations of baseline EEG power for at least 5 s (Roach and Mathalon, 2008; Petrucci et al., 2021).

PEA was defined as a state of repeated electrographic seizure activity in the EEG with interictal periods shorter than 5 min between events (Valenzuela and Benardo, 1995; Ryvlin et al., 2013; Burman et al., 2019). PEA onset was determined by identifying a sustained transition from discrete seizures to continuous or near-continuous spiking, confirmed by reduced variability in interictal interval durations across successive events (Hawkes and Hocker, 2021; Dusing et al., 2024). EEG traces and spectrograms were evaluated to confirm persistent high-frequency activity and loss of return to baseline between events. Mice that entered PEA typically do not recover to their preseizure baseline and remain in this state until death (Devinsky et al., 2016). This state is equivalent to “status epilepticus” in most cases, but we chose to use the broader term PEA due to potential differences between laboratories in criteria used to define status epilepticus in mice (Machnes et al., 2013; Dusing et al., 2024).

### Analysis of EEG/video to assess sleep/wake state

Breathing characteristics can differ between sleep/wake states (active wake, inactive wake, NREM sleep, REM sleep). We used EEG and automated video tracking to distinguish NREM, REM/inactive wake, and active wake so that breathing could be compared between mice during the same state. Because our transmitters are limited to two biopotential leads, we could not record nuchal EMG to distinguish REM from inactive wake, so REM/inactive wake are grouped together. We first identified active wake (movement) using the freezing module in the ezTrack software (Pennington et al., 2019, 2021; RRID:SCR_021362). Timepoints with detected movement were filtered out using the following parameters: mt_cutoff, 7; FreezeThresh, 200; and MinDuration, 75. We did not analyze breathing during movement due to the high variability in breathing parameters. After movement filtering, sleep/wake states were scored in 10 s epochs across 6 h blocks using the Spike2 software (Spike2 script sleepscore 706). Sleep states were determined based on EEG and EMG frequency characteristics and validated post hoc using standard criteria (Buchanan and Richerson, 2010; Buchanan et al., 2015; Smith et al., 2018). REM/inactive wake was defined by moderate-amplitude, moderate-frequency EEG activity in the 4.5–8 Hz range. NREM sleep was identified by high-amplitude, low-frequency EEG (0.5–4 Hz) with minimal or no EMG activity (Brodbeck et al., 2012; Kloefkorn et al., 2022).

### Analysis of diaphragm EMG to measure breathing

Raw diaphragm EMG signals were high-pass filtered at 60 Hz and then rectified and integrated using a 36–50 ms window to minimize electrocardiogram (ECG) artifacts (Zhan et al., 2010). From the rectified and integrated trace, we calculated peak amplitude, inspiratory duration, area under the curve, and instantaneous breathing frequency for each inspiratory burst (Jensen et al., 2019).

Apneas (spontaneous) were defined as pauses where the respiratory cycle length (period) was ≥2.5 times the mean of the preceding 10 breaths within the same behavioral state (Nakamura et al., 2003; Marcouiller et al., 2014). All apneic events were verified manually in diaphragm EMG traces. Postsigh apneas (Bastianini et al., 2019), defined as being within 8 s of a sigh, were identified separately and excluded from spontaneous apnea counts. Sighs were identified as breaths with peak amplitudes at least twice the average of the preceding 10 breaths within the same sleep/wake state (Wilhelm et al., 2001). All sigh and apnea events were manually verified by inspecting diaphragm EMG traces (raw, filtered, and then rectified and integrated) in Spike2.

Breathing frequency and diaphragm EMG parameters were measured from one 1–3 min EMG segment each hour, starting 90 h prior to PEA onset until death during periods of stable breathing. In addition, one 1–3 min EMG segment was selected for analysis at each stage of disease progression: 0, 1, 2–3, 4–5, 6–10, and 11–20 seizures per day, early PEA (within 2 h of PEA onset), and late PEA (within 2 h of death). Stages 0, 1, 2–3, 4–5, and 6–10 include 10 mice. The 11–20 seizures per day and early PEA stages include nine mice because one PTEN–cKO died before reaching these stages (animal B). The late PEA period includes seven mice because two additional mice died between early and late PEA. EMG amplitude values were normalized within each animal to the average sigh amplitude measured during the preseizure baseline to account for differences in strength of EMG signal due to electrode placement and allow us to compare EMG amplitude between mice.

### Histology

Immunohistochemical analysis was used to characterize the expression of tdTomato in Tg^CamKIIα-Cre/+^; *ROSA^PnP-tdTomato/+^* mice (Jimenez-Ruiz et al., 2018). Adult animals were anesthetized with pentobarbital (0.1 mg/g, i.p.) and perfused transcardially with phosphate-buffered saline (PBS) followed by 4% paraformaldehyde (PFA). Brains were postfixed in PFA for 24 h and cryoprotected sequentially in 15 and 30% sucrose solutions at 4°C. The tissue was embedded in OCT compound, frozen, and sectioned at 20 μm using a cryostat. Sections were stained with rabbit anti-RFP (1:25000; Rockland #600-401-379; RRID:AB_828391) and goat anti-ChAT (1:400; Millipore #AB144P; RRID:AB_2079751) primary antibodies and labeled using Cy3-conjugated donkey anti-rabbit (1:500; Jackson ImmunoResearch Laboratories #711-165-152; RRID:AB_2307443) and Alexa Fluor 488-conjugated donkey anti-goat (1:500; Jackson ImmunoResearch Laboratories #705-545-147; RRID:AB_2336933) secondary antibodies. DAPI (1:60,000; Sigma-Aldrich D9542-1MG; RRID:AB_2869624) was used to counterstain nuclei. Slides were imaged using a confocal microscope, and tdTomato expression was evaluated by estimating the percentage of DAPI + nuclei that colabeled with RFP. Expression levels were estimated as follows: “−” (0–10% of cells), “±” (10–50%), and “+” (>50%; Vuong et al., 2015; Zhou et al., 2023).

### Statistical analysis

Statistical tests from GraphPad Prism (RRID:SCR_002798) were used to analyze the data. Statistical tests used and confidence intervals or median differences are described in Table 1. Two-way ANOVAs, mixed-effect ANOVAs, and unpaired *t* tests were used for data that passed the Shapiro–Wilk test for normality. A Mann–Whitney test was used for nonparametric data. Statistical significance was set at an alpha level 0.05, and the variance in figures is reported within the figure legends. Data from male (six PTEN–cKO, six control) and female mice (four PTEN–cKO, three control) were pooled for analysis of breathing parameters. Prior studies found no differences in seizure frequency or duration in male versus female mice (Aiba and Noebels, 2015; White et al., 2020).

**Table 1.** Summary of statistical analyses

Source data	Structure	Statistical test	*p* value	95% CI difference or median of differences
Figure 2*F*	Parametric	Two-way RM ANOVA	*p* = 0.114	–0.2301 to 2.305
Figure 3*D*	Parametric	Two-way mixed-effect model RM ANOVA	*p* = 0.001	−1.906 to −0.5418
Figure 3*E*	Parametric	Two-way mixed-effect model RM ANOVA	*p* = 0.0150	−5.339 to 0.1843
Figure 3*F*	Parametric	Two-way mixed-effect model RM ANOVA	*p* = 0.31	−5.609 to 1.439
Figure 3*G*	Parametric	Two-way mixed-effect model RM ANOVA	*p* = 0.67	−11.72 to 3.760
Figure 5*A*	Parametric	Two-way mixed-effect model RM ANOVA	*p* < 0.0001	−0.3933 to 0.1926
Figure 5*B*	Parametric	Two-way mixed-effect model RM ANOVA	*p* < 0.001	−0.1082 to 0.009531
Figure 5*C*	Parametric	Two-way mixed-effect model RM ANOVA	*p* < 0.0001	−1.828 to 1.654
Figure 5*D*	Parametric	Two-way mixed-effect model RM ANOVA	*p* < 0.001	−0.01195 to 0.02178
Figure 5*E*	Parametric	Two-way mixed-effect model RM ANOVA	*p* < 0.001	−0.004573 to 0.007081
a	Parametric	Two-way mixed-effect model RM ANOVA	*p* = 0.55	−1.541 to 0.6352
b	Parametric	Two-way mixed-effect model RM ANOVA	*p* = 0.94	−0.2082 to 0.4471
c	Nonparametric	Wilcoxon matched-pair signed–rank test	*p* = 0.99	0.03145
d	Nonparametric	Wilcoxon matched-pair signed–rank test	*p* = 0.75	−0.02015
e	Nonparametric	Wilcoxon matched-pair signed–rank test	*p* = 0.31	0.02941
f	Nonparametric	Wilcoxon matched-pair signed–rank test	*p* = 0.50	0
g	Parametric	Unpaired *t* test	*p* = 0.46	−0.08105 to 0.1681
h	Parametric	Unpaired *t* test	*p* = 0.96	−0.3847 to 0.3943
i	Parametric	Two-way mixed-effect model RM ANOVA	*p* = 0.08	−0.0006625 to 0.2085
j	Parametric	Two-way mixed-effect model RM ANOVA	*p* = 0.71	−0.03664 to 0.2284

## Results

### Forebrain-specific recombination using the *Tg^CamKIIα-Cre^* mouse line

**Figure 1.**
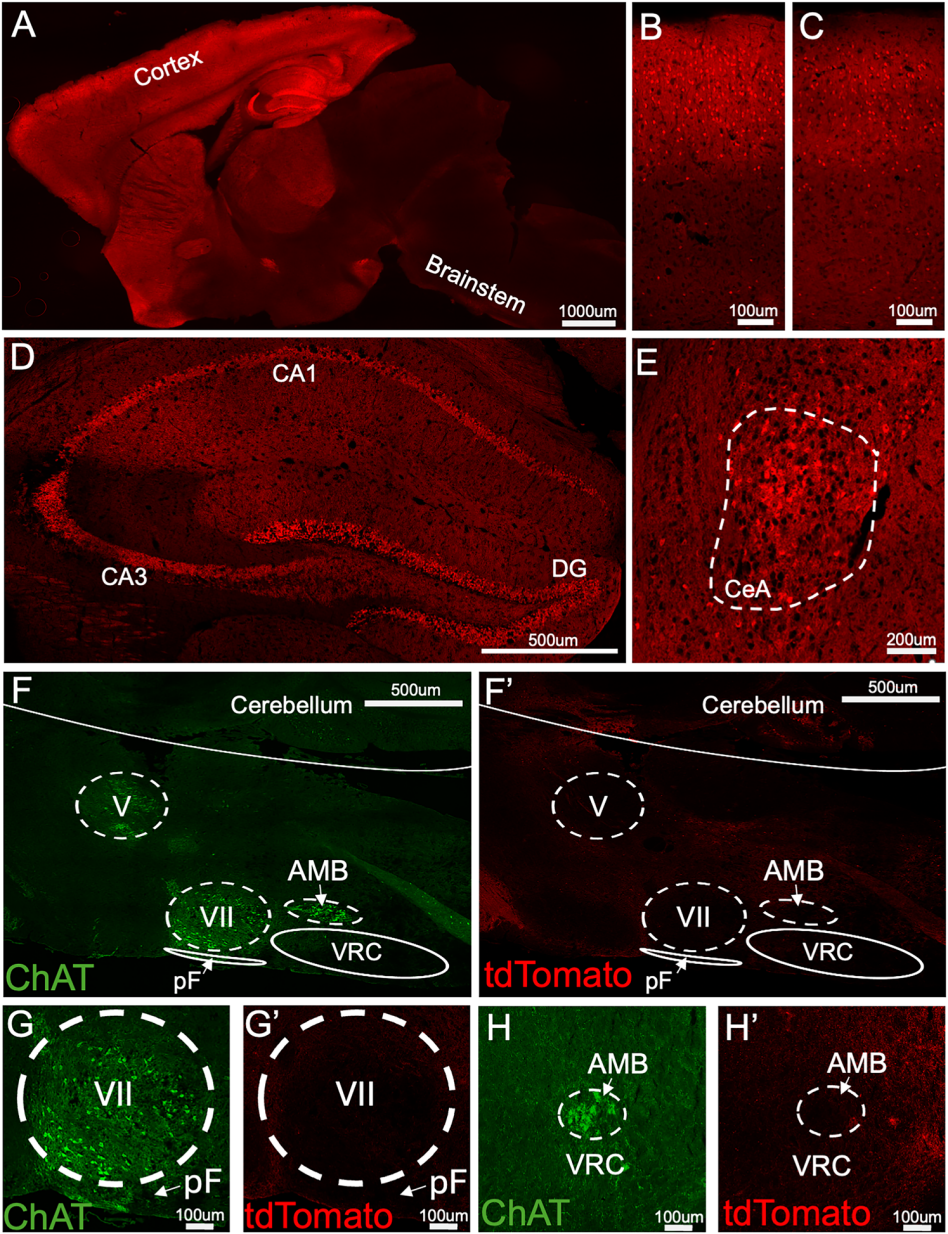
*Tg^CamKIIα-Cre^^/+^*;* ROSA^PnP-tdTomato/+^* mice exhibit widespread recombination in forebrain but not brainstem neurons. Immunohistochemistry for tdTomato was used to label cell bodies and projections of recombined neurons in sagittal or coronal brain sections from adult *Tg^CamKIIα-Cre/+^*;* ROSA^PnP-tdTomato/+^* mice. ***A***, Representative sagittal brain section showing widespread tdTomato expression, with the highest intensity in forebrain regions and minimal signal in the brainstem. ***B***, ***C***, Sagittal sections of the somatosensory (***B***) and motor (***C***) cortices showing tdTomato-labeled cell bodies and processes. ***D***, In the hippocampus, tdTomato + cells were visible in the dentate gyrus, CA1, and CA3. ***E***, The CeA contains tdTomato-expressing somata and processes. ***F***, Antibodies to ChAT were used to mark the trigeminal (V), facial (VII), and nucleus ambiguous (AMB) motor nuclei in sagittal brainstem sections in order to locate the parafacial (pF) respiratory group and ventral respiratory column (VRC). ***F*’**, The same sections from ***F*** show a lack of tdTomato signal in the ventral brainstem, specifically the VRC region. ***G***, ***G*’**, ChAT marked the VII motor nucleus in a sagittal brainstem section and showed an absence of tdTomato signal within the pF region. ***H***, ***H*’**, tdTomato expression in a coronal section of the caudal brainstem was not detectable in the ventral respiratory column at the level of the AMB.

Previous studies have used the CamKIIα-Cre allele to delete PTEN postnatally in the forebrain, leading to seizures and ultimately death (McMahon et al., 2012; Sperow et al., 2012; White et al., 2020). To verify the regional specificity of Cre activity in CamKIIα-Cre mice, we assessed tdTomato expression in *CamKIIα-Cre*; *ROSA^PnP-tdTomato/+^* reporter mice (*N* = 5). In these mice, tdTomato expression indicates successful Cre-mediated recombination and serves as a proxy for PTEN deletion. As shown in Figure 1*A*, tdTomato expression was widespread in the forebrain, with strong labeling in cortical and limbic structures and minimal expression in the brainstem, consistent with prior studies (Tsien et al., 1996). Midsagittal brain sections revealed dense tdTomato fluorescence in cell bodies and processes throughout the forebrain, including the cerebral cortex, and hippocampus (Fig. 1*A*). Higher-magnification images confirm robust expression in somatosensory (Fig. 1*B*) and motor cortices (Fig. 1*C*), as well as across multiple hippocampal subregions, including the dentate gyrus, CA1, CA3, and dorsal subiculum (Fig. 1*D*). These data using a reporter line are consistent with our prior studies showing reduced PTEN protein, elevated phosphorylated AKT (a downstream target of PTEN signaling), and elevated protein synthesis in the cortex and hippocampus of PTEN–cKO mice compared with controls (White et al., 2020). Recombination was also observed in neurons in the central amygdaloid nucleus (CeA; Fig. 1*E*). Prior reports demonstrated that the *Tg^CamKIIα-Cre^* line drives recombination in the CeA (Vincent et al., 2013). Neurons in this region are predominantly inhibitory (Yu et al., 2023), but we did not confirm their neurotransmitter phenotype here. Our analysis confirms prior studies showing that the CamKIIα-Cre mouse line drives recombination in the forebrain, predominantly but not exclusively in excitatory neurons (Tsien et al., 1996; Vincent et al., 2013; White et al., 2020).

Prior studies have not fully characterized recombination (or lack thereof) in the brainstem of CamKIIα-Cre mice. We found very few tdTomato-expressing cells in most of the brainstem, including nuclei critical for respiration. A summary of tdTomato expression across brain areas is detailed in Table 2. Antibodies to choline acetyltransferase (ChAT; a marker of motor nuclei) were used to identify the parafacial respiratory group (ventral to the VIIth motor nucleus) and ventral respiratory column (ventral to the nucleus ambiguous) in sections of the brainstem (Fig. 1*F*–*H*). The ventral respiratory column contains the Bötzinger complex, pre-Bötzinger complex, rostral ventral respiratory group, and caudal ventral respiratory group. However, we did observe recombination in some cell bodies in the lateral spinal trigeminal and cuneate nuclei. We also observed recombination in the dorsal horn of the spinal cord, consistent with endogenous expression of CamKIIα in this region (Larsson, 2018). These findings confirm that CamKIIα-Cre–driven recombination is largely restricted to neurons in forebrain structures and does not occur in brainstem regions responsible for respiratory rhythm generation.

**Table 2.** Expression of tdTomato in *Tg^CamKIIα-Cre/+^*; *ROSA^PnP-tdtomato/+^* mice

Brain region	Presence of RFP cells
Forebrain	
Olfactory tubercle	+
Amygdalohippocampal area	−
Basomedial amygdaloid nucleus	+
CeA	+
Medial amygdaloid nucleus	±
Bed nucleus of the stria terminalis	+
Lateral hypothalamus	−
Paraventricular hypothalamus	−
Hypothalamic nuclei	−
Motor cortex	+
Somatosensory cortex	+
Hippocampus CA1	+
Hippocampus CA2	±
Hippocampus CA3	+
Hippocampus dentate gyrus	+
Hippocampus dorsal subiculum	+
Midbrain	
Periaqueductal gray	−
Locus ceruleus	−
Hindbrain	
Cerebellar cortex	±
Dorsal raphe nucleus	−
Caudal raphe	−
Kölliker–Fuse nucleus	−
Parabrachial nucleus	−
Reticular formation	−
Basal pons	−
Facial nucleus	−
Trigeminal motor nucleus	−
Spinal trigeminal nucleus, lateral	±
Vestibular nuclei	−
Inferior olive	−
Hypoglossal nucleus	−
Caudal ventral respiratory group	−
Ventral respiratory column	−
Bötzinger complex	−
Postinspiratory complex	−
Parafacial respiratory group	−
Pre-Bötzinger complex	−
Retrotrapezoid nucleus	−
Parafacial zone	−
Cuneate nucleus	±
Area postrema	−
Other	
Spinal cord dorsal horn	+

Immunohistochemistry was used to detect tdTomato expression and assess the distribution of tdTomato + neurons throughout the brain in *Tg^CamKIIα-Cre/+^*; *ROSA^PnP-tdTomato/+^* mice. Expression was evaluated by estimating the percentage of DAPI-labeled nuclei that colabeled with RFP and categorized as follows: “−” (0–10% of cells), “±” (10–50%), and “+” (>50%). Robust tdTomato expression was observed across multiple forebrain regions, including the cortex, hippocampus, amygdala, and bed nucleus of the stria terminalis. In contrast, minimal or no labeling was observed in brainstem regions critical for respiratory control. TdTomato + neurons were detected in other subcortical regions, including the cerebellum and dorsal horn of the spinal cord (*N* = 5 mice).

### Simultaneous recording of diaphragm EMG and cortical EEG in PTEN–cKO mice

**Figure 2.**
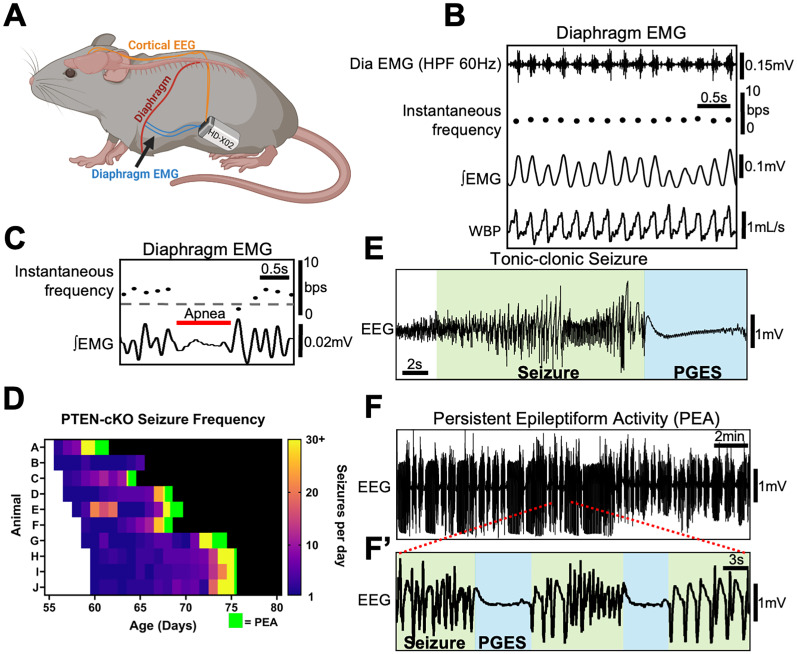
Simultaneous recording of diaphragm EMG and cortical EEG in PTEN–cKO mice. Telemetry devices were implanted in PTEN–cKO mice to monitor respiratory and seizure dynamics across disease progression via diaphragm EMG and cortical EEG recordings. ***A***, Schematic of telemetry implantation showing biopotential leads in the diaphragm and motor cortex. ***B***, Comparison of diaphragm EMG signal to ventilation signal obtained by whole-body plethysmography [top, high-pass (60 Hz) filtered diaphragm EMG; second, instantaneous breathing frequency (breaths per second; bps, black dots); third, rectified and integrated diaphragm EMG (∫EMG); bottom, whole-body plethysmography (WBP), with inspiration represented by an upward deflection of trace]. ***C***, Rectified and integrated diaphragm EMG trace illustrating a spontaneous apnea (red bar). Dots represent the instantaneous breathing frequency. A gray-dotted line denotes breathing frequency threshold for an apnea. ***D***, Heatmap showing seizure frequency over time in 10 PTEN–cKO mice, organized by the age at which each mouse first experienced a seizure. Preseizure (white), PEA (green), and death (black) are also indicated. ***E***, Cortical EEG showing an example of a seizure and postictal generalized EEG suppression (PGES) following the seizure. ***F***, Cortical EEG trace showing seizure activity during PEA (***F*’**) A magnified trace of EEG signal shown in ***F***.

To examine how seizure activity and breathing patterns evolve over the lifespan in an epilepsy model with spontaneous seizures, we used implanted transmitters to continuously measure cortical EEG and diaphragm EMG while video was recorded to assess movement in 10 PTEN–cKO mice in their home cage. We also measured cortical EEG and diaphragm EMG in nine littermate controls. As illustrated in Figure 2*A*, biopotential leads were implanted into the diaphragm and motor cortex to enable simultaneous recording of diaphragm contraction (inspiration) and cortical activity. The diaphragm EMG signal was rectified and integrated to measure the amplitude of individual breaths (peak activity), frequency of inspiration, and duration of inspiration. To validate diaphragm EMG as a reliable measure of breathing frequency and amplitude, we compared the diaphragm EMG signal to concurrently recorded whole-body plethysmography traces. Diaphragm EMG closely tracks inspiratory flow signals detected by plethysmography and accurately detects individual breaths (Fig. 2*B*). Simultaneous measurements of respiratory frequency using diaphragm EMG and whole-body plethysmography showed less than a 1% difference in controls animals^c^ (*n* = 3; mean differences = 0.017, 0.014, and 0.0061 breaths/s) and PTEN–cKO mice before seizure onset^d^ (*n* = 3; mean differences, 0.0067, 0.014, and 0.0037 breaths/s). Importantly, this signal also allows reliable detection of spontaneous apneas or abnormally long periods between breaths, which we define as ≥2.5 times the respiratory frequency (Fig. 2*C*). EEG signal was used to detect seizures (Fig. 2*E*), as well as periods of postictal generalized EEG suppression (PGES), throughout the life of PTEN–cKO mice and monitor their frequency of seizures until they exhibited PEA (Fig. 2*F*,*F*’) and eventually death. Due to the lack of standard criteria for classifying status epilepticus in mice, we use PEA to describe the status of repeated seizure activity. As PTEN–cKO mice aged, we observed a steady increase in spontaneous seizure frequency across all animals (Fig. 2*D*). At first, isolated seizures occur separated by hours or days. Next, mice experienced a few seizures per day, escalating to many seizures per day and finally transitioning (in 9/10 mice) to PEA. We defined PEA as continuous cortical seizure discharges at least every 5 min with minimal to no recovery of EEG power between seizure events. Note that sleep state determination cannot be made during PEA. Although seizure frequency increased reliably over time in all PTEN–cKO animals, neither the average seizure duration (*p* = 0.68) nor the duration of PGES (*p* = 0.42) changed significantly across disease progression (Fig. 2*D*). Thus, we can use telemetry devices to accurately detect seizures, assess disease progression, and evaluate respiratory function continuously in behaving epileptic mice.

### PTEN–cKO mice show an increased frequency of spontaneous apneas during interictal periods

**Figure 3.**
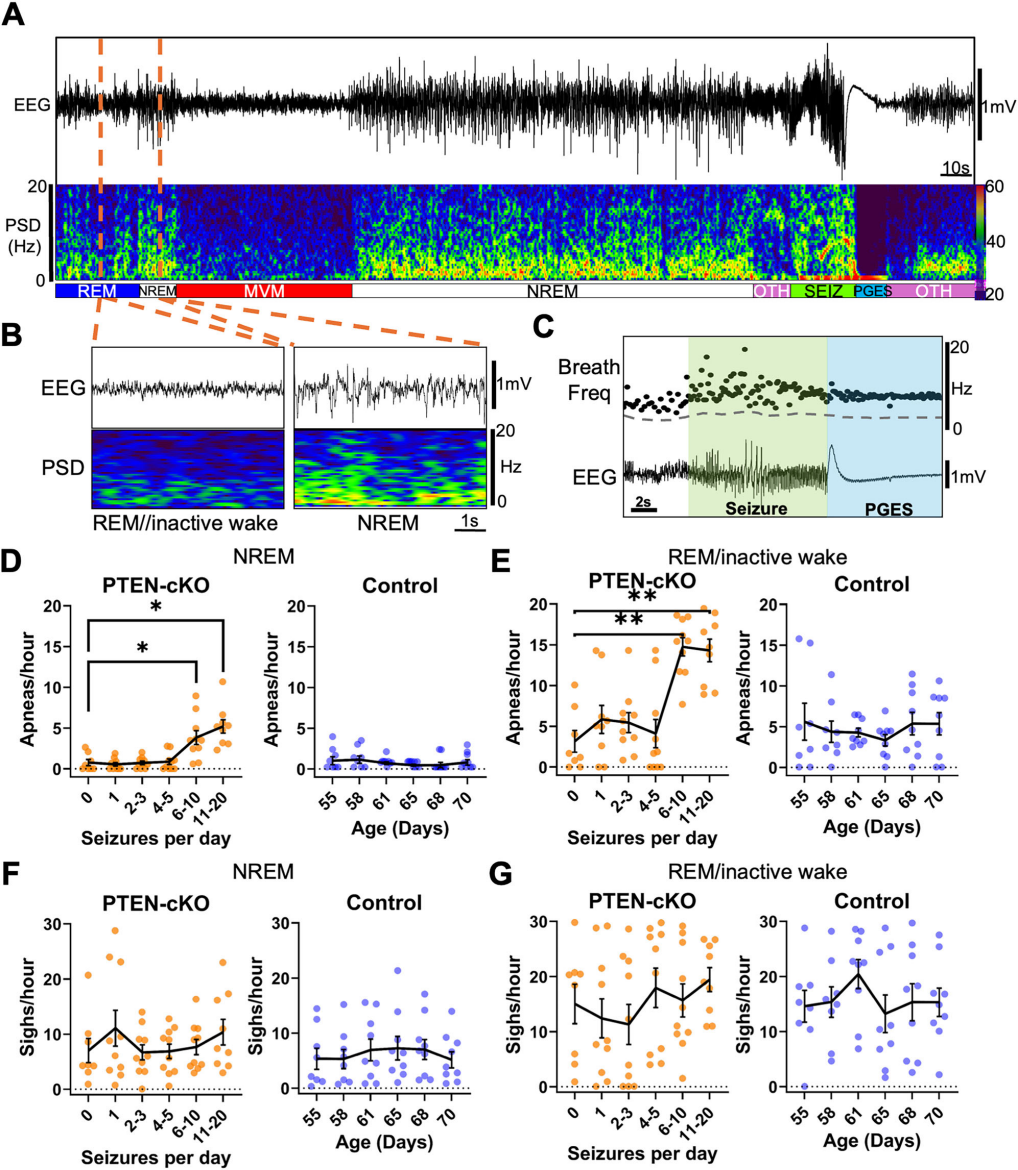
PTEN–cKO mice show an increased frequency of spontaneous apneas during interictal periods. Video, cortical EEG, and diaphragm EMG were continuously recorded from 10 PTEN–cKO and 9 control mice between 7 and 11 weeks of age to assess sleep/wake state and respiratory disruptions during seizures. ***A***, Representative EEG trace (top), corresponding power spectral density (PSD) spectrogram (middle), and classified sleep/wake states (bottom), showing transitions across REM/inactive wake (REM), non-REM (NREM), movement (MVM), seizure (SEIZ), postictal generalized EEG suppression (PGES), and other (OTH). ***B***, Expanded 5 s EEG and PSD traces during REM/inactive wake and NREM sleep. ***C***, Example seizure event showing diaphragm-derived breathing frequency (breaths per second; bps, top) and cortical EEG (bottom); postictal generalized EEG suppression (PGES) follows each seizure. No apneas were detected during this epoch. A gray-dotted line denotes breathing frequency threshold for an apnea. ***D***, ***E***, The average frequency of apneas was assessed during NREM (***D***) and REM/inactive wake (***E***) and plotted for each animal during a 6 h window for each disease stage (seizures per day). CTRL mice, which do not seize, were analyzed over the same time periods as age-matched PTEN–cKO mice. Sigh frequency during NREM (***F***) and REM/inactive wake (***G***) in PTEN–cKO and controls. Data in ***D*** and ***G*** are shown as mean ± SEM. Statistical analysis: two-factor repeated–measure ANOVA with Holm–Sidak post hoc test; *p < 0.05.

To determine how respiratory function is altered by epilepsy, we quantified spontaneous apneas during seizures as well as during interictal periods (between seizures) in PTEN–cKO mice and compared them with the baseline breathing in control mice across the same time span. Video, cortical EEG, and diaphragm EMG were continuously recorded in both groups from 7 to 11 weeks of age (or until death in PTEN–cKO mice). Sleep/wake states were classified using EEG spectral features and video-based motion analysis (Fig. 3*A*,*B*). To assess whether apneas occur in direct association with seizures or PGES, we analyzed 150 total seizure events (Fig. 3*C*) and associated PGES across 10 PTEN–cKO mice. Spontaneous apneas, defined as a breathing period ≥2.5 times the mean cycle length of the preceding 10 breaths, were infrequent during ictal or postictal states in this epilepsy model, occurring in only 14% of seizures and 4% of PGES events. The frequency of apneas during seizures did not differ between early (1–5 seizures/day; 0.07 ± 0.14 apneas per seizure) and late (6–20 seizures/day; 0.12 ± 0.19 apneas per seizure) stages of disease progression^e^. Similarly, apnea frequency during PGES events remained unchanged between early (0.10 ± 0.22 apneas per event) and late (0 apneas per event) stages^f^. The average duration of apneas was 0.49 ± 0.10 s during seizures and 0.47 ± 0.08 s during PGES events, with no significant changes across disease stages for either seizures^g^ or PGES events^h^. Breathing frequency was typically irregular during seizures, as shown in Figure 3*C*. Thus, unlike some other epilepsy models [e.g., *Depdc5* (Kao et al., 2023), *Scn8a* (Wenker et al., 2022), DBA-1 (Marincovich et al., 2021), etc.], this model exhibits irregular breathing with only rare and brief apneas during seizures.

We next quantified apnea frequency and duration during interictal periods. To compare PTEN–cKO mice at similar levels of disease progression, data were stratified by seizure frequency (0, 1, 2–3, 4–5, 6–10, or 11–20 seizures per day). Control mice, which did not exhibit seizures, were grouped using age-matched bins corresponding to the seizure frequency distributions observed in PTEN–cKO animals. We analyzed apnea frequency during NREM, where breathing is typically most regular, separately from apnea frequency during REM/inactive wake periods. Note that postsigh apneas were not included in this analysis of apneas. Apnea frequency significantly increased in PTEN–cKO mice during NREM (Fig. 3*D*) and REM/inactive wake (Fig. 3*E*) at disease stages in which daily seizure counts exceeded six. Controls showed no stage-related change (Fig. 3*D*,*E*). Similarly, apnea duration during NREM^i^ and REM/inactive wake^j^ showed no significant group differences. Sigh frequency also did not change during NREM or REM/inactive wake across these stages in PTEN–cKO mice or controls (Fig. 3*F*,*G*). These findings demonstrate that during both NREM sleep and REM/inactive wake periods, PTEN–cKO mice experience an increase in spontaneous apneas during interictal periods in parallel with an increase in seizure frequency.

### PTEN–cKO mice exhibit slower, deeper breathing as epilepsy progresses

**Figure 4.**
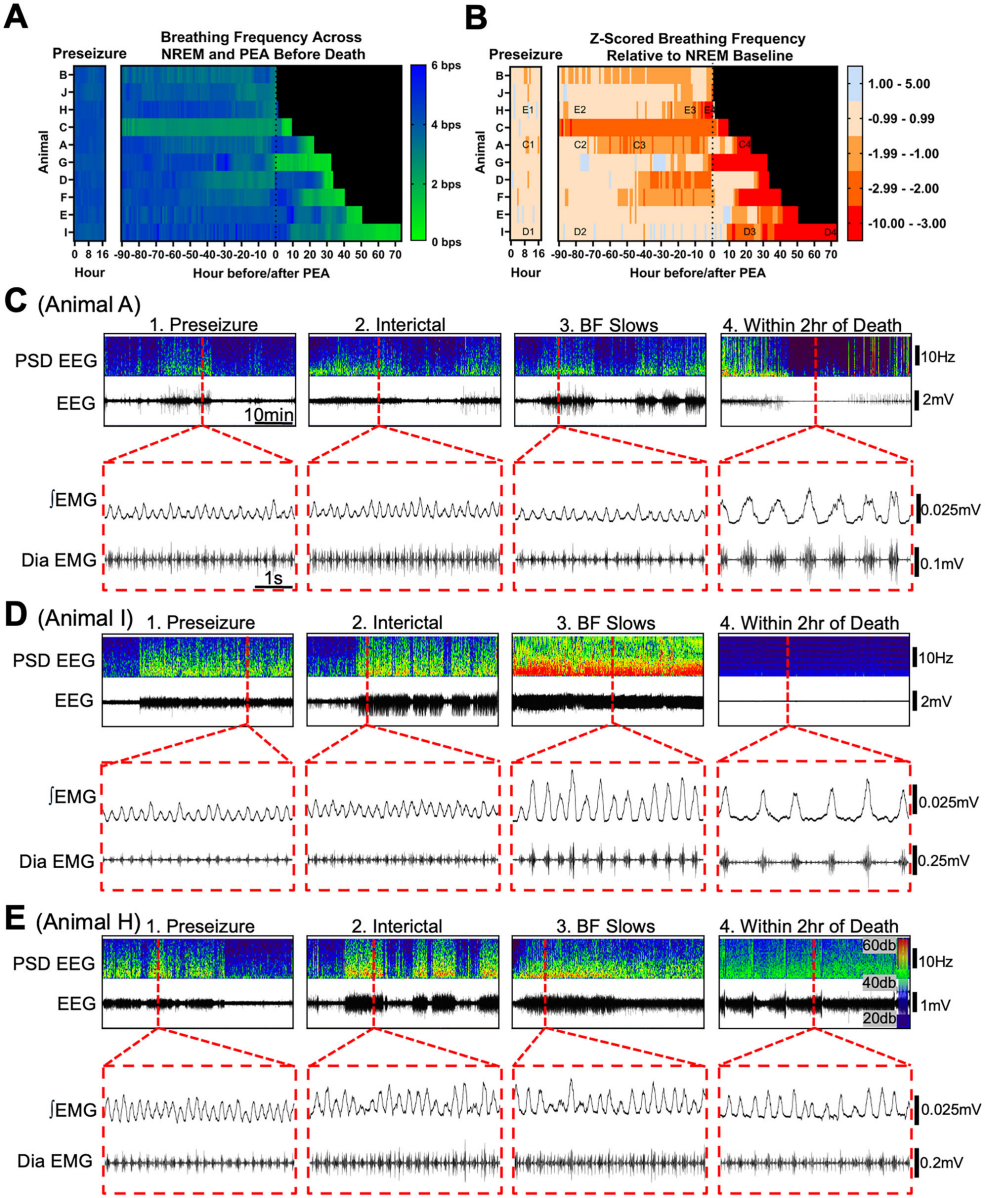
PTEN–cKO mice exhibit distinct changes in diaphragm activity patterns as epilepsy progresses. Continuous diaphragm EMG and cortical EEG recordings were obtained from 10 PTEN–cKO mice (the same Animals A–J shown in Fig. 2*D*) with breathing frequency and amplitude of diaphragm EMG were analyzed. ***A***, Breathing frequency, in breaths per second (bps), was sampled over a 1 min period of rest (either NREM sleep or PEA and no movement) each hour. The heatmap shown here illustrates the frequency over days prior to PEA onset until death for Animals A–J. Note that Animal B did not enter PEA, so Time 0 is death. Before the detection of the first seizure, respiratory frequency (left) is shown over 16 h to show the consistency of the respiratory rate. ***B***, Heat map showing breathing frequency *z*-scores (number of standard deviation units away from the mean) calculated from the breathing frequencies shown in ***A*** by comparing each 1 min sample to the same animal's average breathing frequency during a 6 h preseizure baseline period. Letters on the graph indicate time points of traces shown in ***C***, ***D***, and ***E*** for selected animals. ***C–E***, Representative EEG and diaphragm EMG traces from three PTEN–cKO mice selected to illustrate breathing trajectories across disease progression at four time points: preseizure, during NREM (interictal), after breathing slowed by at least one standard deviation (BF slows), and late PEA (within 2 h of death). Each time point includes a 1 h trace of EEG signal as well as the derived power spectral density (PSD EEG) to illustrate cortical activity surrounding the selected examples of diaphragm activity (5 s each). Red-dotted lines denote the time point of each diaphragm EMG signal shown below each EEG trace. Both 60 Hz filtered (Dia EMG) and rectified and integrated (∫EMG) diaphragm EMG signals are shown. ***C***, Animal A shows a slowing of the respiratory rate 2 d prior to PEA. As the animal approaches death, the diaphragm inspiratory bursts are much less frequent and of higher amplitude, and their duration is longer than preseizure breathing. ***D***, Animal I shows a slowing of the respiratory rate, an increase in diaphragm amplitude, reduced cortical EEG power, and longer inspiratory duration shortly after entering PEA. ***E***, Animal H shows a slowing of breathing and increased amplitude and duration of inspiratory bursts beginning prior to PEA.

We next analyzed changes in breathing frequency and amplitude over disease progression in PTEN–cKO mice. We observed consistent slowing of breathing at late stages of disease progression in all mice, although the timing and rate of decline varied across individual mice (Fig. 4*A*). In 8/10 animals, breathing frequency was consistently lower than the preseizure frequency by at least one standard deviation prior to the animals entering PEA (Fig. 4*B*). In the other two animals, breathing frequency declined by at least one standard deviation after they entered PEA but >24 h prior to death. In most cases (8/10), breathing slowed at least two standard deviations (or more) below preseizure frequency for hours or days prior to death. As breathing slowed, animals also showed an increase in diaphragm amplitude and duration of inspiratory bursts, consistent with deep and/or labored breathing (Fig. 4*C*–*E*). All seven PTEN–cKO mice that remained in PEA for at least 10 h exhibited these large increases in the amplitude and duration of diaphragm activity prior to death. These mice also showed reduced cortical EEG power between seizures which resembled PGES, even when the interval between seizures lasted >5 min (Fig. 4*C*,*D*). Thus, severe changes in the pattern of breathing as epilepsy progresses are evident from diaphragm EMG recordings, with changes most often occurring prior to the onset of PEA.

**Figure 5.**
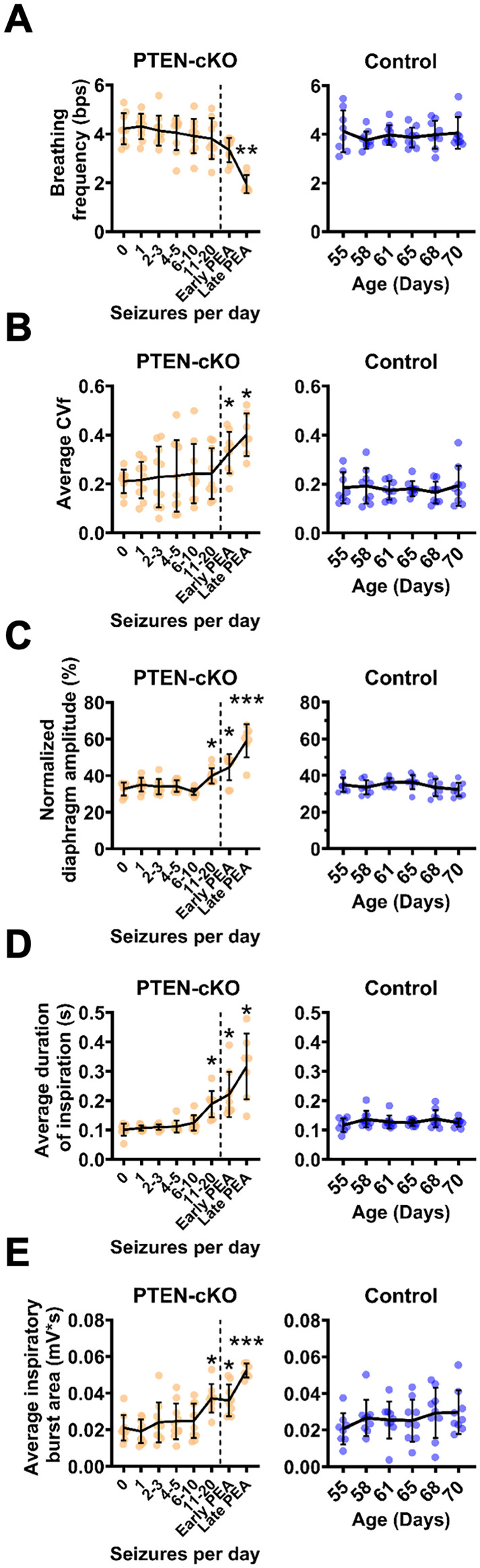
Breathing frequency and diaphragm activity change over disease progression in PTEN–cKO mice. Continuous recordings of cortical EEG were used to assess seizure frequency and PEA, and the diaphragm EMG signal was used to measure respiratory parameters during 1–3-min-long epochs over different stages of seizure progression in PTEN–cKO mice as well as control mice over the same time period. The breathing parameters measured by EMG include (***A***) breathing frequency (breaths per second; bps), (***B***) CVf, (***C***) normalized diaphragm EMG amplitude, (***D***) inspiratory duration, and (***E***) rectified/integrated inspiratory burst area. Data are presented as mean ± SD. Age and seizure group comparisons were analyzed using a two-way mixed-effect repeated–measure ANOVA. *p < 0.05; **p < 0.01; ***p < 0.001 using Tukey's multiple-comparison test comparing each stage of disease progression to preseizure stage (0 seizures per day). Stages 0, 1, 2–3, 4–5, and 6–10 include 10 mice. The 11–20 seizures per day and early PEA stages include nine mice, while the late PEA period includes seven mice.

We further characterized deterioration of respiratory function across the course of disease by analyzing multiple respiratory parameters in PTEN–cKO mice during NREM stratified by seizure burden as well as early (within 2 h of onset) and late stages (within 2 h of death) of PEA. We analyzed 1–3 min segments of NREM sleep or PEA to assess respiratory frequency and inspiratory effort through five key parameters: (1) breathing frequency, (2) coefficient of variation of frequency (CVf), (3) normalized EMG amplitude, (4) inspiration duration, and (5) the inspiratory burst area (Fig. 5). We selected NREM as opposed to REM/inactive wake stage for these analyses because breathing is more regular during NREM. The respiratory rate was significantly slower at early PEA (*p* = 0.004) and late PEA (*p* = 0.02) compared with preseizure in PTEN–cKO mice and was significantly influenced by genotype and disease stage (mixed-effect model, *p* = 1.7 × 10^−5^; Fig. 5*A*). We next measured the variability in frequency by calculating the CVf for each disease stage. CVf increased significantly during early PEA (*p* = 0.01) and late PEA (*p* = 0.02) compared with preseizure and was significantly influenced by genotype and disease stage (mixed-effect model, *p* = 0.004; Fig. 5*B*). The respiratory rate and CVf of PTEN–cKO mice were not significantly different from controls prior to PEA. These data confirm that the respiratory rate and consistency are maintained during the early stages of disease progression in PTEN–cKO mice but significantly decline as animals enter PEA.

We assessed inspiratory effort in the same segments of NREM or PEA throughout disease progression, in which we analyzed breathing frequency. Diaphragm EMG amplitude was measured for each breath and normalized to each animal's preseizure sigh amplitude to correct for differences in EMG lead placement and allow comparisons of amplitude between mice. A significant increase in diaphragm amplitude was observed beginning at the 11–20 seizure stage (*p* = 0.03) compared with preseizure, which further increased at the late PEA stage (*p* = 1 × 10^−15^). Inspiration duration was significantly longer at the 11–20 seizure stage (*p* = 0.03) compared with preseizure and further increased at the late PEA stage (*p* = 0.04). Finally, the inspiratory burst area, reflecting total diaphragm activity during each breath, was larger at the 11–20 seizure stage (*p* = 0.04) compared with preseizure and further increased at the late PEA stage (*p* = 0.0003). Our results demonstrate that inspiratory effort, as measured by three different parameters characterizing diaphragm activity, is significantly increased at late stages of epilepsy progression but prior to animals entering PEA. Moreover, there is a further increase in inspiratory effort in late PEA consistent with deep, labored breathing.

### Terminal respiratory failure in PTEN CKO mice

**Figure 6.**
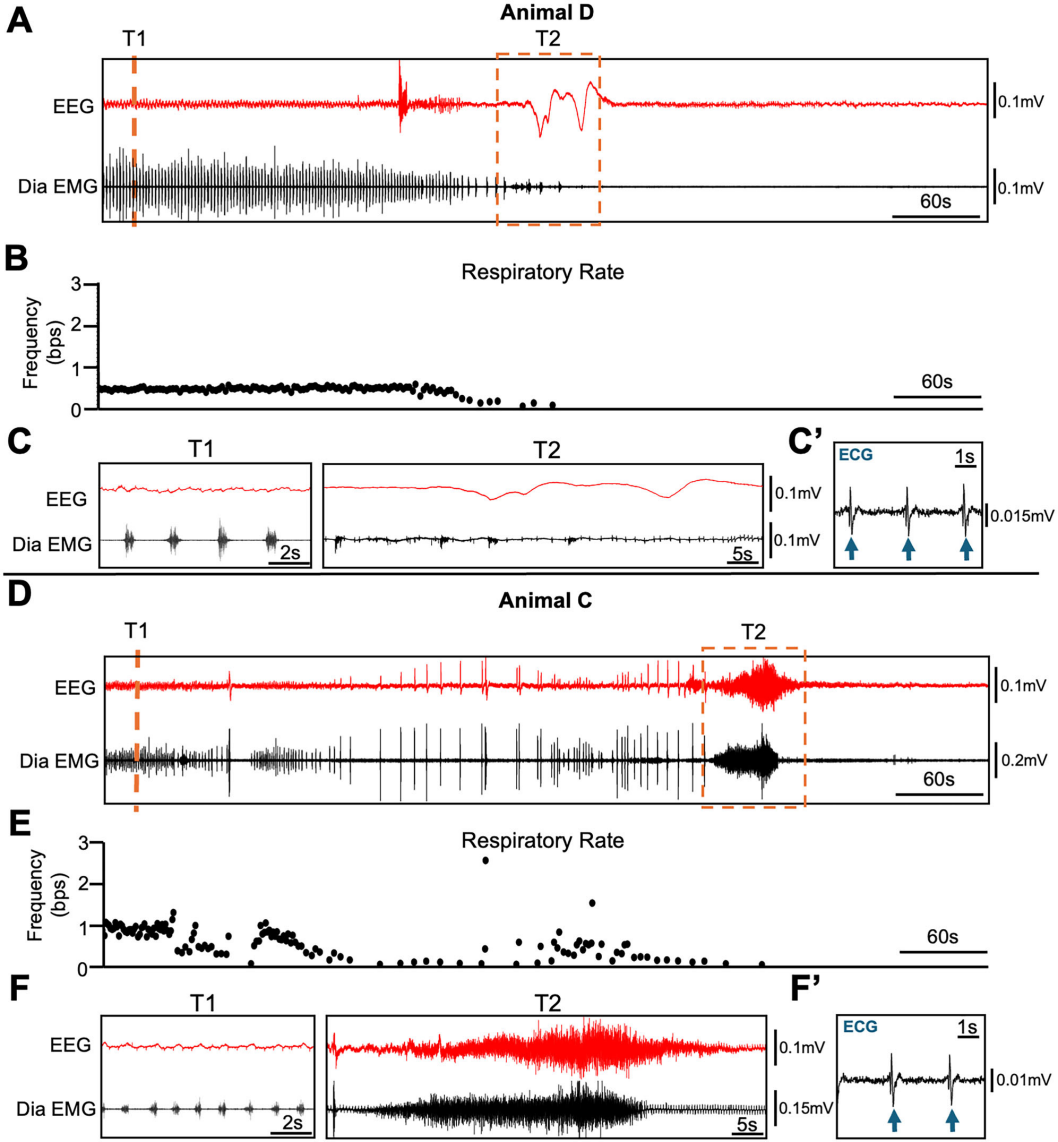
Respiratory failure precedes cardiac arrest in PTEN–cKO mice. Simultaneous EEG and EMG recordings illustrate respiratory failure in two PTEN–cKO mice. ***A***, Representative 10 min traces of cortical EEG (red) and 60 Hz high-pass filtered diaphragm EMG (black) from Animal D. Vertical orange dashed lines mark time points T1–T2 shown in ***C***. ***B***, Instantaneous respiratory frequency over the same 10 min period shown in ***A***; each dot represents the frequency of a single breath (bps). Note that this mouse averaged 4 bps preseizure. ***C***, Expanded 10 s EEG and Dia EMG traces corresponding to T1–T2 in ***A***: T1, regular low-frequency breathing, and T2, final inspiratory burst. ***C*’**, Diaphragm EMG trace following the cessation of breathing shown at larger scale to illustrate ECG signal contaminating EMG. Blue arrows indicate persistent cardiac electrical activity after respiratory arrest. ***D***, Ten-minute EEG and Dia EMG traces from Animal C, which displayed a terminal tonic burst of diaphragm activity. ***E***, Instantaneous respiratory frequency over the same 10 min period is shown in ***D*** for Animal C. Note that this mouse averaged 4 breaths/s preseizure. ***F***, Expanded EEG and Dia EMG traces from T1 to T2 as marked in ***D***. Time points correspond to T1, consistent low-frequency breathing, and T2, terminal tonic burst in Dia EMG. ***F*’**, A high-resolution 5 s trace following the cessation of breathing for Animal C showing raw diaphragm EMG and ECG contamination (blue arrows), indicating persistent cardiac electrical activity after respiratory arrest.

We examined diaphragm activity, brain activity (EEG), and ECG activity (a contaminating signal detectable in our diaphragm EMG traces prior to filtering) to ascertain the cause of death in PTEN–cKO mice. In all 10 animals, terminal apnea (e.g., no more inspiratory bursts detected by diaphragm EMG) preceded terminal asystole (e.g., cessation of ECG signal). Brain activity (EEG power) also decreased after or at the time of cessation of breathing and prior to terminal asystole in all 10 mice. In the time leading up to terminal apnea, 4/10 animals showed a decline in respiratory frequency until breathing ceased (Fig. 6*A*–*C*). Other mice (4/10) showed an irregular breathing pattern, including prolonged apneas, just prior to respiratory failure (Fig. 6*D*–*F*). Some animals (8/10) showed an unusually prolonged period of diaphragm contraction (Fig. 6*D*), consistent with tonic phase apnea, at/near the time of respiratory failure. Thus, PTEN–cKO mice consistently exhibited respiratory failure prior to cardiac failure and a loss of EEG power, indicating brain death.

## Discussion

This study demonstrates that mice with forebrain-specific PTEN deletion exhibit progressive respiratory dysfunction that emerges alongside seizure development and intensifies with disease progression, ultimately culminating in terminal respiratory failure. Through continuous cortical EEG and diaphragm EMG recordings, we observed that respiratory disturbances escalated as PTEN–cKO mice aged and spontaneous seizure frequency increased. These disturbances included increased interictal apnea frequency, slower and more variable breathing rates, prolonged inspiratory durations, and, ultimately, complete cessation of breathing. This pattern of progressive respiratory decline aligns with clinical and preclinical observations of altered breathing during and between seizures (Dlouhy et al., 2015; Dhaibar et al., 2019; Somboon et al., 2019; Bravo et al., 2021) and suggests that forebrain seizure activity may contribute more substantially to respiratory failure in epilepsy than previously appreciated (Nobis et al., 2019).

SUDEP is defined as the sudden death of a person with epilepsy, without a structural or toxicological cause identified at autopsy, and excluding status epilepticus (persistent seizures). It is the leading cause of epilepsy-related mortality, and mounting evidence suggests that respiratory dysfunction is a primary contributor (Ryvlin et al., 2013; Kim et al., 2018; Vilella et al., 2019a). Although 9/10 PTEN–cKO animals would not meet the clinical definition of SUDEP since they died during PEA, the sequence of respiratory dysfunction observed here, including respiratory failure preceding cardiac arrest, parallels key aspects of SUDEP (Ryvlin et al., 2013; Devinsky et al., 2016; Shan et al., 2022).

Our analyses of a Cre-reporter line confirmed that CamKIIα-Cre recombination was largely confined to the forebrain, with minimal expression in brainstem respiratory centers. This expression pattern is consistent with previous reports of CamKIIα promoter-driven recombination in neurons in the cortex, hippocampus, and amygdala (Tsien et al., 1996; Butt et al., 2021; Veres et al., 2023). Our previous work using this PTEN–cKO mouse line confirmed loss of PTEN protein in forebrain neurons (White et al., 2020). Notably, we observed recombination in the dorsal horn of the spinal cord and lateral part of the spinal trigeminal nucleus, so we cannot rule out an effect on breathing due to altered sensory function. However, it seems unlikely that sensory deficits would cause the observed respiratory deficits, especially considering that we only detected respiratory deficits after the onset of seizures, and the deficits became more severe as seizure frequency increased. Thus, one or more of the forebrain structures (i.e., the cortex, amygdala, hippocampus, etc.) that undergo recombination are likely to be the initial source of circuit dysfunction leading to altered breathing.

Increased apnea frequency, longer inspiratory duration, and increased peak diaphragm amplitude were the earliest signs of respiratory decline. Although other studies have found that a decrease in the variability of respiratory frequency, likely reflecting poor adaptability, could predict respiratory decline under some conditions (Brack et al., 2002; Rolland-Debord et al., 2023), we did not observe changes in the variability of breathing frequency until after they entered PEA, when their breathing became both slow and irregular. Prior studies in both animal models and people with epilepsy showed that increased variability of breathing during interictal periods could be predictive of more severe postictal hypoxemia (Purnell et al., 2017; Dhaibar et al., 2019; Sainju et al., 2023). The increased variability of breathing and slow, deep breathing pattern indicate that PTEN–cKO animals at late stages may be hypoxemic, but we did not test this directly.

It was notable that apneas during the ictal or postictal window were infrequent in PTEN–cKO mice, despite the fact that all 150 seizure events analyzed in this study that occurred prior to PEA were generalized tonic–clonic seizures at 5 or above on the Racine scale. Unlike other epilepsy models in which apneas cluster ictally or postictally (Marincovich et al., 2021; Bacq et al., 2022; Wenker et al., 2022), PTEN–cKO mice exhibit a progressive rise in brief interictal apneas, but rare peri-ictal apneas prior to PEA. Previous studies have linked PGES events, particularly those with extended durations, to SUDEP risk (Lhatoo et al., 2010; Vilella et al., 2019b). In the PTEN–cKO model, we observed PGES after every seizure event analyzed prior to PEA with no change in PGES duration and only rarely observed apneas. We do not know the reason for the more consistent presence of PGES in these mice compared with patients with focal epilepsy, although it may be due to the widespread (throughout the cortex, hippocampus, and parts of amygdala) loss of PTEN in our model, compared with humans with somatic mutations restricted to focal regions. We did not observe many apneas during PGES in these mice, but that does not rule out a role for PGES in SUDEP as there are likely multiple mechanisms that can lead to SUDEP. We did not measure EEG or perform direct current recordings in the brainstem, and thus we cannot rule out the possibility that PGES and/or spreading depolarizations can spread from the forebrain to the brainstem and could contribute to breathing abnormalities and death.

The mechanisms underlying the progressive respiratory dysfunction observed in our study are likely multifactorial. PTEN is a key negative regulator of the PI3K/mTOR pathway, and its deletion alters neuron morphology, leads to aberrant neuronal migration, increases neuronal excitability, disrupts synaptic homeostasis, and alters axonal projections (Fraser et al., 2008; Sperow et al., 2012; Tariq et al., 2022; Yonan and Steward, 2023). These changes are likely to contribute to abnormal network dynamics and may directly impact forebrain circuits responsible for modulating breathing. However, PTEN loss has also been associated with impaired inhibition and heightened seizure susceptibility, which may propagate pathological activity to subcortical regions that are not directly impacted by the PTEN mutation (Chen et al., 2019). In addition, recurrent seizures are expected to further disrupt brain function by inducing neuroinflammation, causing neuronal damage or death, and reshaping synaptic connectivity (Aiba and Noebels, 2015; Rosch et al., 2018; Jansen et al., 2019; Khan et al., 2024; Hollis and Lukens, 2025). These secondary changes could impact forebrain structures that modulate breathing or potentially spread to regions of the mid/hindbrain that control breathing. Thus, although numerous direct connections between the forebrain and brainstem respiratory centers (Schottelkotte and Crone, 2022) might be directly impacted by loss of PTEN function, we cannot rule out that the effects on respiratory function may be indirectly caused by intermediate targets of forebrain neurons and/or brainwide dysfunction. Nevertheless, this study highlights the need to more thoroughly investigate the role of forebrain pathways in modulating breathing as well as the spread of dysfunction from the forebrain to the brainstem in the context of epilepsy.

A limitation of this study is that we did not make repeated measurements of metabolic rate or blood gases during seizure progression. For example, experiencing repeated seizures could have pleiotropic effects on mouse physiology, such as a decrease in metabolism, that may indirectly cause slower breathing. Furthermore, the slowed rhythm and increased inspiratory effort (deep or labored breathing) at late stages of disease is consistent with insufficient ventilation and hypoxia, but this was not directly assessed. In addition, because diaphragm EMG cannot assess changes in expiration, airway patency, or air flow, we could not assess with certainty whether the deep breathing at late stages of disease were gasping or agonal breathing, but this would be consistent with the subsequent respiratory failure. Notably, we did not observe an increase in sighs prior to PEA, suggesting that the mice are not hypoxic at this stage. However, we cannot rule out the possibility that the mice experience hypoxia prior to PEA but have another deficit that prevents them from responding appropriately to hypoxia with an increase in sigh frequency.

Another limitation of this study is that we did not include PTEN^+/+^; Tg^CamKIIα-Cre/+^ control mice. Although it is possible that Cre expression could cause adverse effects in forebrain neurons, it is unlikely that this is the cause of respiratory abnormalities because we did not observe apneas or other changes in breathing in PTEN–cKO mice prior to the onset of seizures. None of the control genotypes exhibited seizures in our previous work with this line (White et al., 2020). Notably, we observed that control animals lacking Cre expression, as well as PTEN–cKO animals prior to seizure onset, exhibited baseline respiratory rates near or above 200 bpm, which is higher than typically reported for adult mice using plethysmography (Friedman et al., 2004). However, it should be noted that we recorded and analyzed the respiratory rate in mice housed in their home cage, as opposed to using a small plethysmography chamber that restricts movement. Thus, even though we only analyzed periods during which the animal is asleep and/or not moving, the animal is free to move around the cage in between periods used for analysis. Other studies have shown higher rates of respiration (250–400 bpm) in mice when monitored over long compared with brief time periods (Receno et al., 2019). Importantly, our control mice show a consistent respiratory rate throughout the time frame used for analysis (Fig. 5*A*), indicating that this is a normal rate for these mice in their home cage.

As seizure frequency increased, we observed respiratory dysfunction in PTEN–cKO mice that we would not have been able to capture without continuous monitoring. The breathing rate slowed markedly, while EMG amplitude, inspiratory duration, and burst area all increased, indicating a deep or labored breathing pattern at late stages of disease. Labored, gasping, and/or agonal breathing (which we did not attempt to distinguish in this study) are signs of respiratory distress (Peña et al., 2008; Tulaimat and Trick, 2017). Importantly, the increase in inspiratory effort was first detectable in the PTEN–cKO group of mice prior to entering PEA, while they were still capable of performing routine tasks during wakeful periods such as feeding, drinking, and exploring the cage. These results suggest a steady decline in respiratory function as opposed to a sudden collapse. Furthermore, our study suggests that evidence of increased inspiratory effort, including during sleep, could potentially be a predictor of SUDEP in epilepsy patients.

Our results also raise the question of whether insufficient ventilation contributes to more rapid seizure progression. Although severe hypoxia can induce seizures (Fukushi et al., 2020, 2023; Wolff et al., 2020; Ala-Kurikka et al., 2021), it is not clear whether breathing abnormalities can contribute to seizure progression in epileptic individuals. Our results are consistent with a prior study showing that a forebrain restricted mutation in DEPDC5 in excitatory cortical Layer 5 neurons and dentate gyrus neurons led to respiratory dysfunction (ictal apneas in their model) and SUDEP (Kao et al., 2023). These findings reinforce a growing body of evidence implicating forebrain dysfunction and its downstream effects as key contributors to SUDEP.

A critical future direction would be to evaluate whether commonly used antiseizure medications can modify respiratory outcomes and mortality in the PTEN–cKO model. Although our current study did not test this directly, prior work suggests that seizure suppression alone may be insufficient to prevent SUDEP when PI3K/mTOR signaling is dysregulated (Zhang et al., 2013; Koene et al., 2019). One possibility to explain this outcome is that epilepsy causes permanent disruption in the forebrain control of breathing. Additional studies would be necessary to test this hypothesis and identify seizure-independent mechanisms that might contribute to respiratory failure in epilepsy.

In summary, forebrain-specific PTEN deletion in mice results in rapid seizure progression associated with progressive respiratory dysfunction and culminating in respiratory failure. Abnormal respiratory patterns that include increased interictal apneas, reduced breathing frequency, and increased inspiratory effort progressively worsen as seizure frequency increases. Future studies should investigate the circuit-level interactions between the forebrain and brainstem and assess whether targeted interventions, such as mTOR inhibitors, seizure-suppressing therapies, or surgical disruption of specific pathways, can preserve respiratory function and reduce mortality in epilepsy.

## Synthesis

Reviewing Editor: Viji Santhakumar, University of California Riverside Decisions are customarily a result of the Reviewing Editor and the peer reviewers coming together and discussing their recommendations until a consensus is reached. When revisions are invited, a fact-based synthesis statement explaining their decision and outlining what is needed to prepare a revision will be listed below. The following reviewer(s) agreed to reveal their identity: Jamie Maguire.

Summary: The study examines an important and timely question of how cortical seizures impact brainstem function. The question had high relevance for understanding respiratory failure in SUDEP. The authors simultaneously monitored seizure activity and breathing over development in a cortex specific spontaneous seizure model (PTEN cKO mice). Their effort to manipulate an epilepsy associated gene specifically in the cortex and ability to continuously monitor seizures and breathing over the course of disease progression are major strengths of the study. The most valuable finding of this study is that respiratory function deteriorates in parallel with increased seizure burden. Another important finding is that apneic events are most prevalent during the interictal period. Together these observations suggest seizures cause persistent changes in brainstem function. However, the lack of mechanistic discussion of the e findings limits the impact of this work. Additionally, there are some concerns that with the model and relevant controls, EEG and apnea analysis which need to be addressed. Moreover, some of the conclusions including the assertion that this work supports forebrain role in control of breathing during sleep and that PTEN cKO mice are prone to breathing problems particularly during NREM sleep are not fully justified based on the experimental results.

Specific Comments

1) The CeA mainly consists of inhibitory neurons, so it is surprising that CamKIIα reporter mice show dense labeling in that region. The authors should confirm their cre is specific to excitatory neurons.

2) Expression of cre under the CamKIIα promotor is not benign (PMID: 36543864) Therefore it is not appropriate to pool cre only and floxed only mice as a common control.

3) A healthy acclimated adult mouse should have a respiratory feq less than 200 bpm. The high resp. rates in figs 4-5 are surprising. Control and PTEN cKO mice show high pre-seizure respiratory frequency. Respiratory frequencies well over 200 bpm are commonly attributable to stress. Additional controls are needed to rule out the possibility that respiratory freq decreases over development due to acclimation rather than as a consequence of increased seizure burden. Fig. 5A argues against this possibility cre dependent effects need to be ruled out? Do floxed only mice (no cre) show a lower freq?

4) Please define the genetic background of experimental mice.

5) Some measure of PTEN function/expression is need to conform knockout. dT expression can be used to gauge transfection efficiency and specificity but it cannot be used to validate deletion of the target gene.

6) There is some confusion regarding how apneas were measured. The methods section states "Apneas were defined as breaths with durations at least 2.5 times longer than the average of the preceding 10 breaths" whereas the results section states "spontaneous apneas, which appear as periods of absent breathing and a sudden drop in instantaneous breathing frequency". This should be clearly defined since this is a major focus of the manuscript.

7) The effort to differentiate spontaneous apnea from post-sigh apnea is a strength. However, it is surprising that sigh related activity was not analyzed, especially since this will increase with hypoxia which presumably is the underlying cause of altered respiratory activity with disease progression. This additional analysis will strengthen the study.

8) The authors use the term "persistent epileptiform activity (PEA)" which they define as continuous cortical seizure discharges at least every 5 minutes with minimal to no recovery of EEG power between seizure events. This is not a standard term in the field and it should be stated how this differs from status epilepticus.

9) It is surprising that seizure freq or PEA did not correlate with PGES, especially since PGES are considered a mechanism of SUDEP. It would be helpful if the authors could discuss mechanisms specific to their model that might minimize occurrence of PGES or limit spread of depolarizing block. On a related note, if apnea did not correlate with PGES, then that implies PGES fail to propagate to the brainstem. Can the author use these results to address the relevance of PGES as a basis for SUDEP? If they think its model specific, some mechanistic rationale is needed.

10) As the authors note, respiratory failure in epilepsy is generally thought to occur during the ictal or postictal period, so it is surprising that was not the case in this mode. This needs some mechanistic explanation. For example, maybe the spectral profile of seizures in this model differs from other models? Alternatively, did apnea correlate with seizure severity based on the Racine scoring system?

11) Please include detailed discussion on how repeated seizures might impact brainstem function. Does seizure burden alter metabolism? For example, decreased breathing freq may be a normal adaptive response if metabolic activity decreases in this model?

12) Decades of heart rate literature show that high hr variability (as measured by entropy) correlates with good health, whereas a drop in variability is indicative of a cardiac event. The same has been shown to be the case for respiratory freq. Therefore, it is surprising that CVf increases in PTEN cKO. Please provide a more detailed explanation.

13) Gasps are typically large amplitude, short duration breaths with characteristically fast rise time. This does not appear to be the case in Fig. 4C-E. Please provide additional details/support for the claim this is gasping and how this relates to the human condition.

14) The repeated assertion that PTEN cKO mice are prone to breathing problems particularly during NREM sleep requires a comparison of respiratory function during other sleep-wake states (namely REM or wake). That was not done. The authors should consider measuring apneas during wakefulness rather than just during NREM or revising the statement to be consistent with the experiments that were conducted.

15) The conclusion that this work "adds to the growing evidence that the forebrain exerts significant influence over brainstem respiratory networks, particularly during sleep when top-down regulation is critical" is not adequately substantiated. It is true that forebrain-brainstem projections provide an anatomical substrate by which seizure activity may descend to the brainstem to disrupt breathing. However, the forebrain is thought to provide volitional, emotional and arousal influences on control of breathing, all of which are expected to be minimal during sleep conditions evaluated in this study.

16) The figures would benefit from more clear labeling and description of what is being represented.

a. Figure 2B: labels for what each trace is depicting would be helpful. It is in the figure legend, but labeling in the figure itself would be useful.

b. Figure 2E: the persistent seizure activity is not clear. A magnified trace would help.

c. Figure 3C: a marker for apnea threshold would be useful.

d. Figure 4: clearly indicate what different row represent (C-E). In Fig. 4E the EEG shows no activity, is this PGES?

## Author Response

Response to Reviewers Specific Comments:

1) This study uses a reporter mouse to show CamKIIa expression is restricted to the forebrain (fig. 1). However, these results are in stark contrast to a previous study that showed high levels of CamKIIa in the mouse brainstem (PMID: 23632380). The authors should make some effort to reconcile these disparate observations. This is important because all conclusions of this study are based on CamKIIa being absent from the brainstem.

For our study, we selected a mouse line that did not have significant recombination in the brainstem (except as noted in the Results section) and validated the expression in Figure 1 and Table 1. In addition, Dr. Tonegawa and his colleagues (Tsien et al., 1996) demonstrated in Figure 4E of their manuscript a lack of recombination in the brainstem using the same CamKIIα-Cre 29-1 line with a different reporter line, which we now explicitly acknowledge in the results section (Lines 264-265). Thus, although the CamKIIα-GFP transgenic mouse line described in PMID: 23632380 appears to have expression in the medulla, the CamKIIIα-Cre line (T29-1) used in this study does not. It is common for different randomly integrated transgenic mouse lines, even those using a similar promoter, to differ in their expression patterns- most likely due to differences in their integration site. This is also noted in the paper by Dr. Tonegawa and his colleagues as they generated several lines from the same CamKIIα-Cre construct that each had somewhat different expression patterns.

2) Related to point #1, based on the Allen Mouse brain ISH dataset (https://www.proteinatlas.org/ENSG00000100150-DEPDC5/brain) DEPDC5 is also robustly expressed in the mouse medulla. Therefore, the claim "These findings suggest that forebrain dysfunction alone, even in the absence of intrinsic brainstem defects, may be sufficient to impair breathing" is not accurate.

We revised our claim to state, "These findings demonstrate that forebrain dysfunction can lead to impaired breathing, consistent with our results using PTEN-cKO mice." (Lines 63-64). Although it is true that Depdc5 transcripts are present in the medulla, our reference is to a study that uses a conditional deletion strategy (similar to the one we used) to delete Depdc5 gene expression exclusively in the cortex using a Rbp4-Cre mouse line and Depdc5 floxed alleles (Kao et al., 2023). In this mouse line, excitatory cortical neurons lack Depdc5 but medulla expression of Depdc5 is maintained. The specificity of the Rbp4-Cre mouse line for forebrain excitatory neurons is described in a paper by Dr. Sue McConnell's laboratory (Kao et al., 2023). Thus, we believe that the paper by Kao et al., in which the Depdc5 mutation is restricted to the forebrain, supports our new claim. We removed the reference to a human study (Meletti et al., 2024) in which brainstem mutations could not be ruled out (Lines 62-63).

However, there is still some concern about the model system and high respiratory rate reported here. It is possible that, as authors propose, that high metabolic activity caused by increased locomotom might contribute tyo a high normal respiratory freq.

We added to the results section a comparison of respiratory rate measured simultaneously by diaphragm EMG and plethysmography in multiple animals to further validate our methods (Lines 308-312). Our study used continuous home-cage recordings to measure breathing and the rates we report for control and pre-epileptic PTEN cKO mice (~4-4.5 breaths/s) fall within typical values obtained with plethysmography (3-5 breaths/s) as well as approaches used in freely behaving mice (3.3-7 breaths/s) (Receno et al., 2019). Although respiratory rates can vary in mice due to activity level, genetic background, and environmental conditions, we observed consistent and stable respiratory rates over days/weeks in control animals (Fig. 5A). Consistent with our hypothesis that mice are more active in their home cage than when movement is restricted by a plethysmography chamber, we observed a higher respiratory frequency in the home cage compared to a plethysmography chamber (4.0 {plus minus} 0.7 vs. 3.1 {plus minus} 0.1 breaths/s; paired t-test p = 0.0132; N=6) when measured in the same mice.

Kao H-Y, Yao Y, Yang T, Ziobro J, Zylinski M, Mir MY, Hu S, Cao R, Borna NN, Banerjee R, Parent JM, Wang S, Leventhal DK, Li P, Wang Y (2023) Sudden Unexpected Death in Epilepsy and Respiratory Defects in a Mouse Model of DEPDC5-Related Epilepsy. Annals of Neurology 94:812-824.

Meletti S, Duma GM, Burani M, Danieli A, Giovannini G, Osanni E, Micalizzi E, Mambretti F, Pugnaghi M, Vaudano AE, Bonanni P (2024) Ictal and Postictal Central Apnea in DEPDC5-Related Epilepsy. Neurology Genetics 10:e200183.

Receno CN, Eassa BE, Cunningham CM, DeRuisseau LR (2019) Young and middle-aged mouse breathing behavior during the light and dark cycles. Physiol Rep 7:e14060.

Tsien JZ, Chen DF, Gerber D, Tom C, Mercer EH, Anderson DJ, Mayford M, Kandel ER, Tonegawa S (1996) Subregion- and Cell Type-Restricted Gene Knockout in Mouse Brain. Cell 87:1317-1326.
